# Giant unruptured sinus of Valsalva aneurysm successfully managed with valve-sparing procedure – a case report

**DOI:** 10.1186/s13019-020-1061-1

**Published:** 2020-01-08

**Authors:** Miklós Pólos, Cristina-Maria Șulea, Kálmán Benke, Bence Ágg, Attila Kovács, István Hartyánszky, Béla Merkely, Hans-Joachim Schäfers, Zoltán Szabolcs

**Affiliations:** 1 Heart and Vascular Center, Semmelweis University, Budapest, Hungary; 2 George Emil Palade University of Medicine, Pharmacy, Science and Technology of Targu Mures, Targu Mures, Romania; 3 Department of Thoracic and Cardiovascular Surgery, Saarland University, Medical Center and Saarland University Faculty of Medicine, Saarbrücken, Germany

**Keywords:** Valsalva aneurysm, Cardiac surgery, Valve-sparing procedure, Suture annuloplasty, Aortic root remodeling, case report

## Abstract

**Background:**

Sinus of Valsalva aneurysm (SVA) is an uncommon cardiac anomaly, with an incidence of less than 1% of open heart surgery cases. Its evolution is most frequently silent, being found incidentally or discovered in the event of its acute rupture. Non-ruptured giant SVAs may cause unusual clinical manifestations, as a consequence of their protrusion into the heart chambers or compression of the coronary vessels and are frequently associated with aortic insufficiency of various degrees of severity. The gold standard treatment for SVAs consists of complete replacement of the aortic root and valve. However, in certain cases, valve-sparing procedures may prove to be a more suitable alternative.

**Case presentation:**

A 68-year-old male patient presented with dyspnea as symptom caused by a large (> 5 cm) right sinus of Valsalva aneurysm. The aneurysm was occupying most of the right ventricle and was associated with severe aortic regurgitation. The surgical treatment of the condition involved valve-sparing root reconstruction procedure (remodeling technique), completed with external stabilization of the aortic valve annulus via running suture annuloplasty. Following the uneventful intervention, the patient did well and his status improved. The follow-up transthoracic echocardiography obtained 1 month after surgery showed a fully competent aortic valve with no regurgitation.

**Conclusions:**

Despite complete aortic root and valve replacement being considered the safest approach to large SVAs complicated with aortic insufficiency, valve-sparing procedures should not be overlooked in case of a dilated aortic root with uncalcified aortic valve. Performing valve-sparing by applying a remodeling technique operation completed with annuloplasty reduces aortic valve insufficiency, avoiding side-effects related to implanted valves.

## Introduction

Sinus of Valsalva Aneurysm (SVA) is considered to be an uncommon cardiac anomaly, being found in less than 1% of the cases in cardiac surgery [1]. It can be either congenital or acquired, the former being more prevalent, with an incidence of less than 3.5% of all congenital heart defects [2]. SVA most frequently affects only one of the sinuses and originates predominantly from the right coronary sinus, in approximately 94% of the cases [3].

The typical evolution of SVAs is towards rupturing, thus carrying a very high rate of mortality. The likelihood of a fatal episode occurring has been shown to be correlated with maximum aneurysm size [4]. However, SVAs may often progress asymptomatically, remaining undetected unless rupture occurs. In rare cases, unruptured SVAs may cause unusual structural and functional anomalies, consequently presenting with atypical clinical manifestations, such as right ventricular outflow tract obstruction [5], conduction disturbances [6], myocardial ischemia [7], or even syncope [8]. As reported by Takach et al., around 44% of SVAs were associated with aortic regurgitation of various levels of severity [1].

In selected cases involving small SVAs, a primary closure or patch closure of the aneurysm may be performed [9]. However, the gold-standard treatment for SVAs consists of complete replacement of both the aortic root and valve, also known as Bentall procedure [10]. As stated by Weinreich et al. [11] and Woo et al. [12], in case of an uncalcified native aortic valve, preserving the valve is becoming a preferred alternative to replacement because of the decreased incidence of valve-related side effects. Over the past three decades, heart surgery has encountered a continuous development of valve-sparing procedures, such as the ones established by David [13] and Yacoub [14]. More recently, Schäfers et al. proposed an aortic valve suture annuloplasty technique involving external stabilization of the annulus following the remodeling of the aortic root [15]. Nevertheless, despite these advances, there are few reports in the current published literature proposing valve-sparing technique as a preferred alternative over complete replacement in the treatment of SVA [16].

## Case presentation

### Patient information

A 68-year-old male patient with no significant past medical history was admitted because of progressive shortness of breath (NYHA III-IV) which began 6 months prior to the admission. Transthoracic echocardiography (TTE) revealed an aneurysmal bulge of the right aortic sinus into the right ventricle without obvious rupture. The patient had smoked for more than 50 years, denied any alcohol consumption and took no medication.

### Clinical findings

On TTE, an extensive unruptured SVA was visible, arising from the right coronary sinus of Valsalva and protruding into the right ventricular chamber, significantly comprising the cavity. TTE also revealed prolapse of the right coronary cusp of the aortic valve and severe (grade IV) aortic valve regurgitation on an otherwise uncalcified native tricuspid aortic valve. The left ventricular systolic function was depressed, with an ejection fraction of 40%, presumably as a consequence of the long-standing progression of the aortic valve regurgitation (LVESD 53 mm, LVEDD 62 mm). TTE revealed no other valvular dysfunctions.

### Diagnostic assessment and timeline

**Fig. 1:**
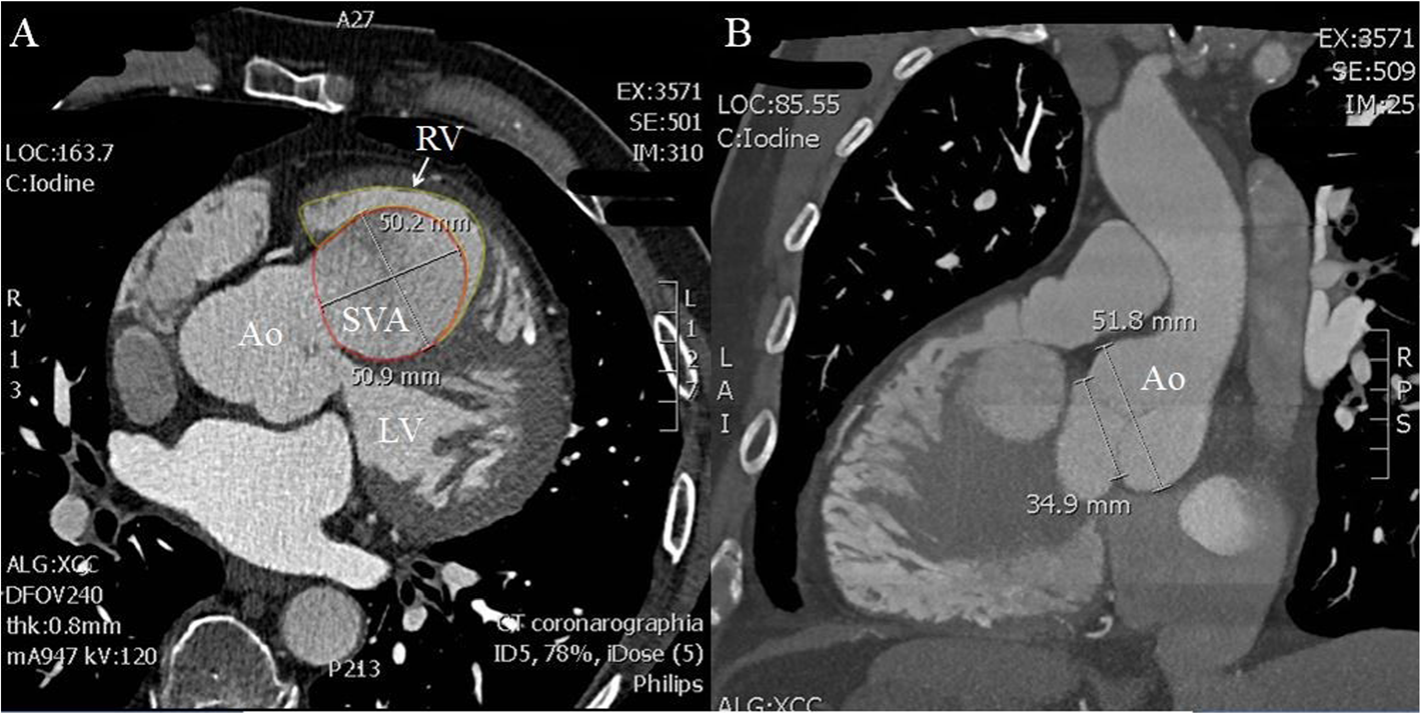
Computed tomography angiography. **a** – Transverse section showing the origin of the SVA from the right aortic cusp, occupying most of the right ventricle (yellow line) which is visible in this incidence as a thin zone around the aneurysm (red line). **b** – Sagittal section showing the diameters of the aortic valve and aortic root at the level of the sinuses of Valsalva. SVA = sinus of Valsalva aneurysm; Ao = aorta; LV = left ventricle; RV = right ventricle

Computed tomography angiography (CTA) confirmed the presence of a saccular aneurysmal structure originating from the right coronary sinus of Valsalva, protruding into the right ventricular chamber, measuring 50,2 mm × 50,9 mm × 64 mm (Fig. 1a). The diameters of the dilated aortic annulus and aortic root measured 34,9 and 51,8 mm, respectively (Fig. 1b). No coronary disease was found. Considering the clinical and paraclinical findings, the patient was scheduled to undergo surgical correction.

### Therapeutic intervention

**Fig. 2:**
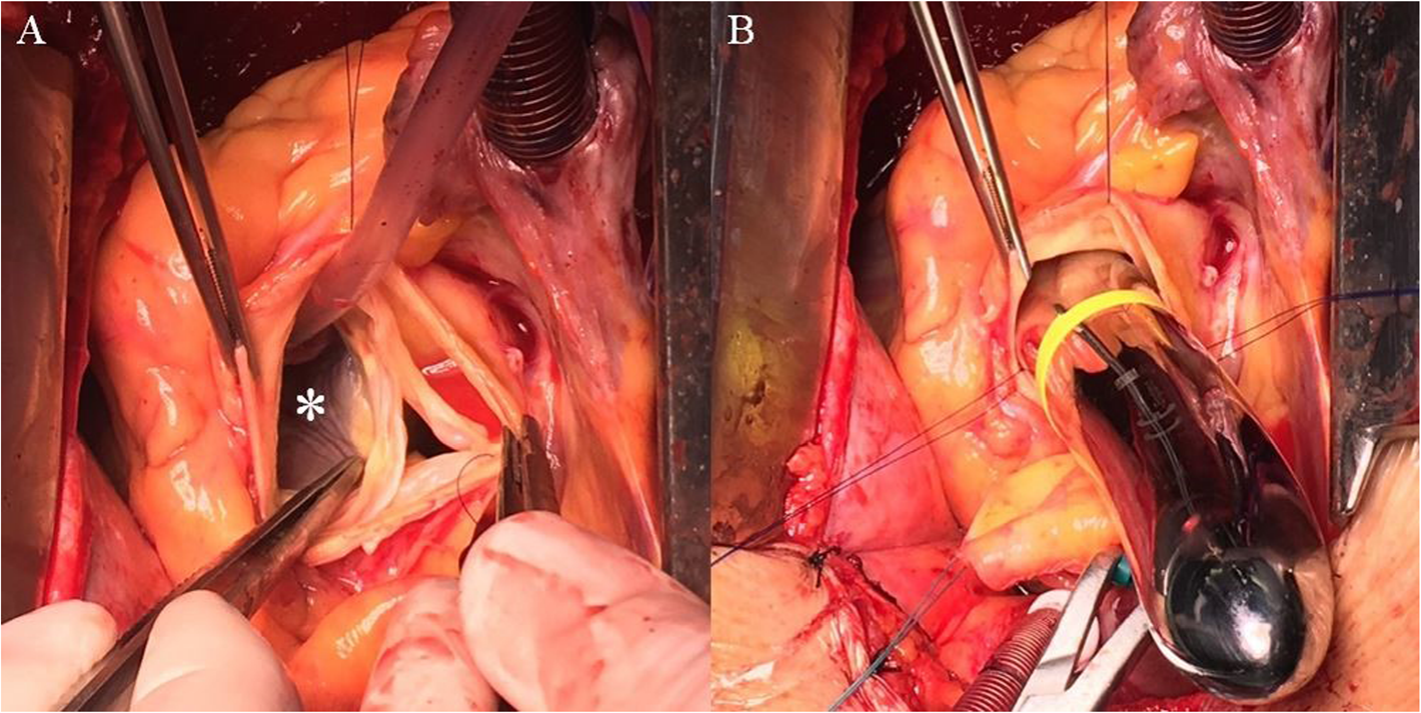
Intraoperative pictures. **a** – Image showing the orifice of the SVA (marked with asterisk) and the dilated aortic root. **b** – Hegar dilator is placed in the orifice of the aneurysm. The image is meant to emphasise the size of the aneurysm, here compared to the size of the 25 mm Hegar dilator which was also used for the suture annuloplasty [16]

**Fig. 3:**
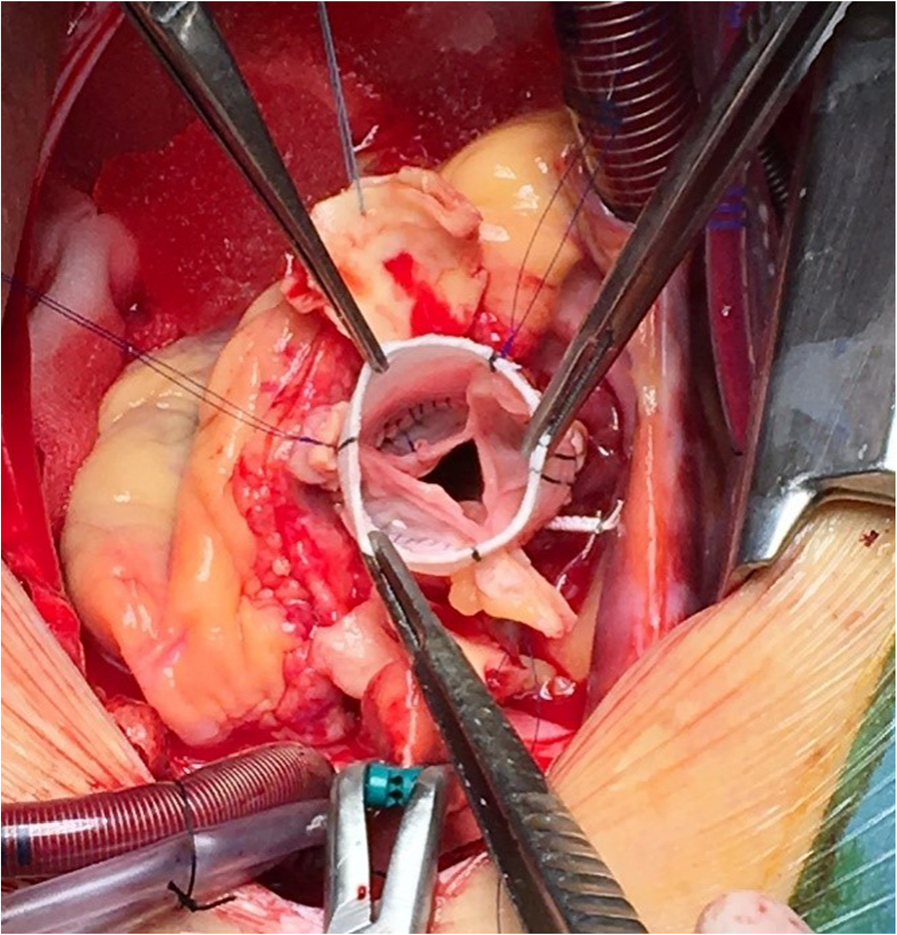
Intraoperative view of the implanted vascular graft, after the exclusion of the aneurysm and the plasty of the aortic valve

As part of the preoperative preparation, because of the depressed LV function, levosimendan was administered. The surgical intervention was performed under general anesthesia. After median sternotomy, cardiopulmonary bypass was applied by cannulating the ascending aorta and the right atrium. Myocardial preservation was obtained via cold Custodiol crystalloid cardioplegia. The aortic valve was exposed using a transverse aortotomy. The intraoperative discoveries confirmed the above findings: a giant aneurysm originating from the right aortic sinus was protruding into the right ventricle (Fig. 2a, b), while the annulus of the aortic valve was severely dilated. However, the aortic valve showed no signs of calcification. Thus, the surgical approach aimed to exclude the aneurysm from the right ventricular chamber and preserve the native aortic valve. The orifice of the saccular aneurysm protruding through the wall of the right ventricle was closed with a running 4–0 Prolene suture. The aortic root was replaced with a 26 mm AlboGraft vascular graft in the manner described by Yacoub et al. (Fig. 3). The aortic annulus was reduced to a diameter of 25 mm by applying the modified basal suture annuloplasty technique proposed by Schäfers et al. [15]. According to the description of the procedure, a running annuloplasty suture was placed at the basal level of the aortic root, thus achieving appropriate external stabilization of the valve. Additionally, free-edge plication of the right and non-coronary leaflets was performed, as well as suture reinforcement of the free margin of the right leaflet, in order to resolve the aortic insufficiency. The effective height of the cusps was corrected to 9 mm [17]. The extracorporeal circulation time was 186 min, while the cross-clamp time was 155 min. After reconstruction, intraoperative control transesophageal echocardiography (TEE) showed competent aortic valve and no significant transvalvular gradient.

### Follow-up and outcomes

The patient’s recovery was favourable. The follow-up TTE obtained 1 month after surgery showed a fully competent aortic valve with no regurgitation.

## Discussion

Sinus of Valsalva aneurysm is defined as an abnormal dilatation of the aortic root situated between the aortic valve annulus and the sinotubular junction. Most of these aneurysms originate from the right sinus of Valsalva and are usually isolated. SVAs may be congenital in origin, caused by the weakness of the elastic and muscular tissues of the aortic wall, as encountered in patients suffering from hereditary connective tissue disorders, such as Marfan or Ehlers-Danlos syndrome [18, 19]. Less often, SVAs may occur as a consequence of degenerative disease (atherosclerosis), infection (tuberculosis, syphilis, infective endocarditis) [20, 21], or aortic dissection, as well as chest trauma [22], or iatrogenic injury [23].

SVAs are highly uncommon, often presenting with silent evolution or unspecific symptoms, such as angina-like chest pain, palpitations, or dyspnea, as in the case presented above. They are frequently detected only as incidental findings or in the event of acute rupture, their most severe and potentially fatal complication. Several cases have been reported in which SVAs caused unusual cardiac manifestations, such as ischemia, conduction disturbances, or right ventricular outflow tract obstruction.

**Fig. 4:**
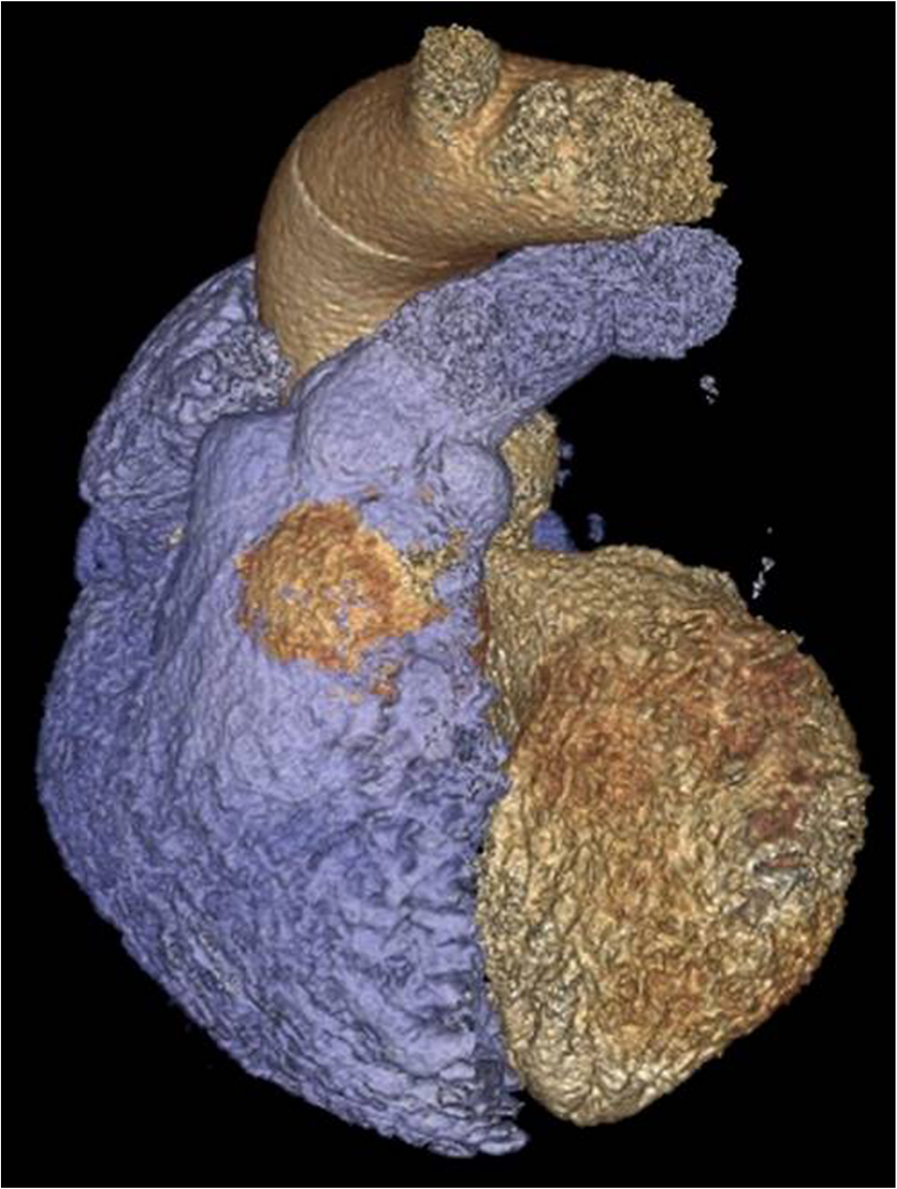
Volume rendering of the CTA scan indicating the SVA in brown colour within the right ventricle in blue

Besides conventional angiography, other noninvasive imaging modalities may prove useful in assessing SVA characteristics, such as TTE, cardiac CTA (Fig. 4), and cardiac magnetic resonance imaging (cMRI). Due to its high sensitivity and wide availability, TTE is the first choice screening modality, helping in accurate characterization of SVAs. In cases such as the one presented, preoperative cMRI would prove most useful in assessing with high sensitivity any complications of the aneurysmal structure, such as rupture or fistulas into the heart chambers.

Levosimendan has been shown to have a beneficial effect on cardiac function, causing fewer side effects than catecholamines. In cardiac surgery, postoperative low cardiac output syndrome (LCOS) may result in multiple organ failure and high mortality. Preoperative administration of levosimendan is recommended in patients with depressed left ventricular function undergoing cardiac surgery because it improves hemodynamics and optimizes postoperative organ function, lowering the risk of LCOS [24].

Regardless of etiology and clinical expression, it is generally agreed that SVAs should undergo surgical correction, despite the surgical treatment modality being a matter of controversy [2]. The safest approach to a giant unruptured SVA complicated with severe aortic insufficiency would be complete replacement of the aortic root and valve. Despite the Bentall procedure being a much more straightforward strategy, valve-sparing techniques are gaining field and we believe that they should not be overlooked in case of an enlarged aortic root but uncalcified aortic valve. Additionally, completed with stabilization via external suture annuloplasty described by Schäfers et al., valve-sparing procedures may offer better outcomes than the classical techniques, considering that optimal stabilization of the aortic annulus is essential for the durability of the repair [15].

## Conclusions

Even in the case of large unruptured SVAs accompanied by severe aortic valve insufficiency, valve-sparing procedures may prove to be feasible. The age and functional status of the patient, as well as the possibility to avoid implanted valve side effects, may prompt repair strategy. Published data about cases of SVAs surgically managed by valve-sparing techniques is scarce. More than that, a giant SVA associated with severe aortic insufficiency surgically managed with a modified suture annuloplasty technique has not been previously reported. Therefore, we present this case hoping that it will encourage surgeons to consider valve-sparing techniques as a real possibility of treatment in such cases.

## Data Availability

Not applicable.

## Abbreviations

cMRI: Cardiac magnetic resonance imaging
CTA: Computed tomography angiography
LCOS: Low cardiac output syndrome
LV: Left ventricle
LVEDD: Left ventricle end-diastolic diameter
LVESD: Left ventricle end-systolic diameter
SVA: Sinus of valsalva aneurysm
TEE: Transesophageal echocardiography
TTE: Transthoracic echocardiography

## References

[1] Takach TJ, Reul GJ, Duncan JM, Cooley DA, Livesay JJ, Ott DA, Frazier O (1999). Sinus of valsalva aneurysm or fistula: management and outcome. Ann Thorac Surg.

[2] Feldman DN, Roman MJ (2006). Aneurysms of the sinuses of valsalva. Cardiol.

[3] Guo DW, Cheng TO, Lin ML, Gu ZQ (1987). Aneurysm of the sinus of Valsalva: a roentgenologic study of 105 Chinese patients. Am Heart J.

[4] Coady MA, Rizzo JA, Hammond GL (1997). What is the appropriate size criterion for resection of thoracic aortic aneurysms?. J ThoracCardiovasc Surg..

[5] John SH (2010). A rare case of unruptured sinus of valsalva aneurysm obstructing the right ventricular outflow tract. J Cardiovasc Ultrasound.

[6] Agarwal P, Jain A, Singh P, Singh H, Geelani M, MehtA V (2018). Unruptured right sinus of Valsalva aneurysm dissecting into Interventricular septum causing complete heart block: can early surgical correction revert rhythm disturbances?. World J Cardiovasc Dis.

[7] Braga Carlos Galvão, Ocaranza-Sánchez Raymundo, Durán-Muñoz Darío, Legarra-Calderón J. José, González-Juanatey José Ramón (2016). Unruptured sinus of Valsalva aneurysm presenting as NSTEMI. Archivos de Cardiología de México.

[8] Matteucci M, Rescigno G, Capestro F, Torracca L (2009). Syncope triggered by a giant unruptured sinus of Valsalva aneurysm. Interact Cardiovasc Thorac Surg.

[9] Banerjee S, Jagasia D (2002). Unruptured sinus of Valsalva aneurysm in an asymptomatic patient. J Am Soc Echocardiogr.

[10] Benke K, Ágg B, Szabó L, Szilveszter B, Odler B, Pólos M, Cao C, Maurovich-Horvat P, Radovits T, Merkely B, Szabolcs Z.Bentall procedure: quarter century of clinical experiences of a single surgeon. J Cardiothorac Surg. 2016;11(1). 10.1186/s13019-016-0418-y PMC4724135 26801237

[11] Weinreich M, Yu P, Trost B (2015). Sinus of Valsalva aneurysms: review of the literature and an update on management. Clin Cardiol.

[12] Woo YJ, Frederick JR (2013). Valve-sparing aortic root replacement and Neochordal repair of complex aortic leaflet pathology for ruptured sinus of Valsalva aneurysm Fistulizing to the right ventricle. Ann Thorac Surg.

[13] David TE, Feindel CM (1992). An aortic valve-sparing operation for patients with aortic incompetence and aneurysm of the ascending aorta. J ThoracCardiovasc Surg..

[14] Yacoub M (1996). Valve-conserving operation for aortic root aneurysm or dissection. Operative Techniques in Cardiac and Thoracic Surgery.

[15] Schneider U, Aicher D, Miura Y, Schäfers HJ (2016). Suture Annuloplasty in aortic valve repair. Ann Thorac Surg.

[16] Akashi H, Tayama E, Tayama K, Kosuga T, Takagi K, Aoyagi S (2005). Remodeling operation for unruptured aneurysms of three sinuses of Valsalva. J Thorac Cardiovasc Surg.

[17] Schäfers HJ, Bierbach B, Aicher D (2006). A new approach to the assessment of aortic cusp geometry. J ThoracCardiovasc Surg.

[18] De Bakey ME, Diethrich EB, Liddicoat JE, Kinard SA, Garrett HE (1967). Abnormalities of the sinuses of Valsalva. Experience with 35 patients. J Thorac Cardiovasc Surg.

[19] Oka N, Aomi S, Tomioka H, Endo M, Koyanagi H (2001). Surgical treatment of multiple aneurysms in a patient with Ehlers-Danlos syndrome. J Thorac Cardiovasc Surg.

[20] Sobrinho JH, Silva MA, Fontes WF, Santos MA, Pontes Junior SC, Silva MV, Rubayo EM, Arnoni AS (1989). Syphilitic aneurysm communicating with an aortic sinus of Valsalva. A case report. Arq Bras Cardiol.

[21] Batiste C, Bansal RC, Razzouk AJ (2004). Echocardiographic features of an unruptured mycoticaneurysm of the right aortic sinus of Valsalva. J Am Soc Echocardiogr.

[22] Greiss I, Ugolini P, Joyal M, Bouchard D, Mercier LA (2004). Ruptured aneurysm of the left sinusof Valsalva discovered 41 years after a decelerational injury. J Am Soc Echocardiogr.

[23] Guler N, Eryonucu B, Tuncer M, Asker M (2004). Aneurysm of sinus of Valsalva dissecting into interventricular septum: a late complication ofaortic valve replacement. Echocardiogr.

[24] Toller W, Heringlake M, Guarracino F, Algotsson L, Alvarez J, Argyriadou H (2015). Preoperative and perioperative use of levosimendan in cardiac surgery: European expert opinion. Int J Cardiol.

